# Pregnancy after Bariatric Surgery: A Nested Case-Control Study of Risk Factors for Small for Gestational Age Babies in *AURORA*

**DOI:** 10.3390/nu13051699

**Published:** 2021-05-17

**Authors:** Zainab Akhter, Nicola Heslehurst, Dries Ceulemans, Judith Rankin, Roger Ackroyd, Roland Devlieger

**Affiliations:** 1 Population Health Sciences Institute, Newcastle University, Newcastle upon Tyne NE2 4AX, UK; nicola.heslehurst@newcastle.ac.uk (N.H.); judith.rankin@newcastle.ac.uk (J.R.); 2 Department of Obstetrics and Gynaecology, University Hospitals Leuven, 3000 Leuven, Belgium; dries.ceulemans@uzleuven.be (D.C.); roland.devlieger@uzleuven.be (R.D.); 3 Department of Surgery, Sheffield Teaching Hospitals, Sheffield S10 2JF, UK; roger.ackroyd@nhs.net

**Keywords:** bariatric surgery, obesity, pregnancy, gestational weight gain, nutrition, fetal growth

## Abstract

Bariatric surgery prior to pregnancy is a significant risk factor for small for gestational age (SGA) babies. This case-control study investigated differences between mothers delivering an SGA baby following bariatric surgery, compared to those delivering an appropriate for gestational age (AGA) baby. Out of 129 babies born to mothers in the AURORA cohort study, 25 were SGA (<10th percentile) and 97 were AGA (10th–90th percentile). Higher gestational weight gain (GWG) was significantly associated with decreased odds of SGA (aOR per kg 0.92, 95% CI 0.85–0.99). According to the Institute of Medicine GWG guidelines, 44% of SGA mothers had ‘inadequate’ GWG compared to 17% of AGA mothers. Nearly half of the mothers had ‘excessive’ GWG yet still gave birth to an SGA or AGA baby. Mothers of SGA babies lost more weight following bariatric surgery (45.6 ± 14.4 kg vs. 39.0 ± 17.9 kg). Women who reported receiving nutritional advice following bariatric surgery were significantly less likely to have an SGA baby (aOR 0.15, 95% CI 0.0.4–0.55). Women with a history of bariatric surgery should be provided with specialized support before and during pregnancy to encourage adequate nutritional intake and weight gain to support healthy fetal growth.

## 1. Introduction

Obesity is the most prevalent medical condition affecting women’s reproductive health today. Maternal obesity is defined as a preconception body mass index (BMI) above 30 kg/m^2^ and is associated with a range of severe health complications for both mother and baby [1,2,3]. As obesity increases worldwide, the demand for bariatric surgery is also rising. Bariatric surgery is the most effective treatment for long-term weight loss, and the majority of patients are women of reproductive age [4]. Undergoing bariatric surgery prior to pregnancy significantly reduces the risk of obesity-related comorbidities such as gestational diabetes, hypertension, and infertility [5,6]. However, bariatric surgery prior to pregnancy has also been linked to maternal nutrient deficiencies and small for gestational age (SGA) babies [7]. Procedures which reduce the absorption of micronutrients such as gastric bypass surgery are associated with over twice the odds of having an SGA baby compared to the general obstetric population [8].

Babies are defined as SGA if their birth weight falls under the 10th percentile for their gestational age [9]. Fetal growth restriction (FGR) refers to the antenatal diagnosis of pathologically small babies who are at risk of adverse health outcomes, often due to placental insufficiency, leading to decreased fetal oxygen and nutrient supply [10]. Fetal growth is largely dictated by nutrient availability which is firstly dependent on maternal nutritional intake during preconception and pregnancy, and secondly dependent on the ability of the placenta to transport these nutrients to the fetus [11]. Babies may also be small due to non-placenta mediated growth restriction which is caused by a structural or chromosomal anomaly, fetal infection, or an inborn error of metabolism [9]. Size for gestational age under the 10th percentile is used as a proxy for FGR; however, around 50–70% of SGA babies are estimated to be constitutionally small with fetal growth appropriate for maternal height and ethnicity [9]. FGR is a major risk factor for perinatal morbidity and mortality; however, constitutionally SGA babies also have an increased risk of adverse outcomes including stillbirth and neurodevelopmental impairment [12,13,14]. These outcomes are significantly improved when SGA identification occurs antenatally to allow clinical surveillance and management [15].

The aim of this study was to examine factors which influence the odds of a mother delivering an SGA baby following bariatric surgery, compared to an appropriate for gestational age (AGA) baby. Any associations could be used prior to or during pregnancy to determine whether a mother with previous bariatric surgery is at high-risk of delivering an SGA baby. Factors investigated included modifiable factors which can be influenced by the mother or antenatal care, and also clinical indicators which are influenced by bariatric surgery. Although history of bariatric surgery significantly increases the risk of delivering an SGA baby, not all mothers will; therefore it would be helpful if women with a higher risk can be identified early. This would inform guidelines for clinical practice, allowing health professionals to adapt their preconceptions and antenatal care accordingly to improve infant health outcomes.

## 2. Materials and Methods

### 2.1. Study Design

This case-control study included babies born from the bAriatric sUrgery Registration in wOmen of Reproductive Age (AURORA) dataset; the study protocol is described in detail elsewhere [16]. The AURORA is a prospective cohort study carried out at eight general hospitals in Belgium which includes women aged 18–45 years who have already had, or will undergo, bariatric surgery. Women participating are followed up with until six months after a subsequent pregnancy. Data collected are a combination of clinical data collected by researchers and self-administered data completed by the participants. Relevant data for this study were extracted in June 2018. The study was approved by the local ethical committees of the participating hospitals, and informed consent was obtained from each of the participants (ClinicalTrials.gov: NCT02515214).

### 2.2. Case and Control Definitions

The birth population of Flanders, Belgium was used as the reference group for categorizing the AURORA babies according to size for gestational age. Percentiles were calculated from the data collected in the Study Centre for Perinatal Epidemiology (SPE) [17]. The 10th and 90th percentiles of infant birth weights for gestational age, specific for child sex, parity, and plurality were used as cut-offs for each size for gestational age grouping. Babies were defined as SGA, and therefore a ‘case’, if the baby’s birth weight was below the 10th percentile. Babies with a birth weight between the 10th and 90th percentile were grouped as AGA and were used as controls. Babies with a birth weight above the 90th percentile were grouped as large for gestational age (LGA) and were excluded from analyses. Exclusions were also made for pregnancies with missing birth weight, or gestational age data.

### 2.3. Maternal Factors

Maternal variables that may impact the size for gestational age were defined a priori and divided into two groups. The first group consisted of modifiable factors: gestational weight gain (GWG), smoking, physical activity, receipt of nutritional advice, and nutritional serum levels. GWG was defined into groups of ‘Inadequate’, ‘Recommended’, or ‘Excessive’ depending on pre-pregnancy BMI according to the Institute of Medicine (IOM) guidelines (Table 1) [18]. These GWG guidelines are considered optimal for fetal growth, in addition to other maternal and perinatal outcomes, in the general obstetric population [19]. Smoking status was defined into ‘Yes’ or ‘No’ categories if any number of cigarettes were smoked per day during the second trimester. Women were categorized into whether or not they actively participated in sports or exercise, not including habitual physical activity at home or work, during the second trimester more than once per month. Mothers answered ‘Yes’ or ‘No’ when asked if they received nutritional advice after bariatric surgery. Serum levels of haemoglobin, red blood cell (RCB) count, iron, folate, vitamin B12, calcium, vitamin D 25-OH, and albumin were documented from second trimester blood samples.

The second group of factors were the potential clinical indicators of SGA: type of surgery, surgery to conception interval, preconception BMI, gestational diabetes, pregnancy-induced hypertension, and weight loss from surgery to conception. For type of surgery, laparoscopic adjustable gastric banding (LAGB) and sleeve gastrectomy (SG) were grouped together, as they both reduce stomach capacity, and there are no changes to other portions of the digestive tract. Roux-en-Y gastric bypass (RYGB) was grouped with biliopancreatic diversion (BPD), as these procedures involve bypassing a portion of the small intestine which is responsible for nutrient absorption. The surgery-to-conception interval was calculated as the interval between the date of surgery and 280 days before the baby’s due date. Preconception BMI (kg/m^2^) was calculated using maternal preconception weight and height. In the analyses, the effect of preconception BMI on size for gestational age was calculated for a 5-unit change in BMI to translate back to the WHO BMI categories which are in approximately 5 kg/m^2^ increments. Gestational diabetes screening was performed at 24 weeks of pregnancy. For initial AURORA participants, gestational diabetes was diagnosed from the oral glucose tolerance test (OGTT), which is now contraindicated in women with a history of bariatric surgery [20]. Newer participants were screened using fasting plasma glucose, HbA1c, a combination of both, or capillary blood glucose measurements (≥5.3 mmol/L fasting glycaemia, ≥7.8 mmol/L one hour after a meal or ≥6.7 mmol/L two hours after a meal) [20]. Gestational hypertension was defined as >140 mmHg systolic blood pressure or <90 mmHg diastolic blood pressure in patients with no known hypertension before pregnancy. Weight loss from surgery to conception was calculated using weight before surgery and preconception weight.

### 2.4. Statistical Analysis

Descriptive statistics for maternal and baby characteristics were reported as frequencies and percentages for categorical variables. For continuous variables, the mean ± standard deviation was given. Categorical variables were investigated using Pearson’s Chi squared (χ^2^) test or Fisher’s exact test. Continuous variables were firstly examined for normality using the Shapiro-Wilk test. Variables that were normally distributed were compared using two-sample *t*-tests, and those which were not were assessed used Mann Whitney-U tests. Logistic regression analysis was used to calculate crude and maternal-age adjusted odds ratios (OR) and 95% confidence intervals (CI) to explore the effect size between SGA and only the a priori defined maternal factors. Sensitivity analyses were carried out including maternal height as a proxy for the baby being constitutionally small to explore if it affected the results. For all analyses, a *p*-value <0.05 was considered statistically significant. However, as bariatric surgery is not common, the study was subject to a limited sample size; therefore, focus was also directed towards trends in the data and clinical significance. Biologically plausible explanations of any differences between the mothers of SGA and AGA babies were reported in the absence of statistical significance, however, with uncertainty about the magnitude of the effect. Missing data (Table S1) were assumed to be missing at random due to factors such as non-attendance at follow up, item non-response in the online questionnaires, or issues with data collection equipment in the clinic; therefore, pairwise deletion was used for the analyses to maximize data availability. All analyses were performed in Stata IC 16.1. The study is reported in line with Strengthening the Reporting of Observational Studies in Epidemiology (STROBE) guidelines [21].

## 3. Results

### 3.1. Maternal and Infant Characteristics

**Figure 1:**
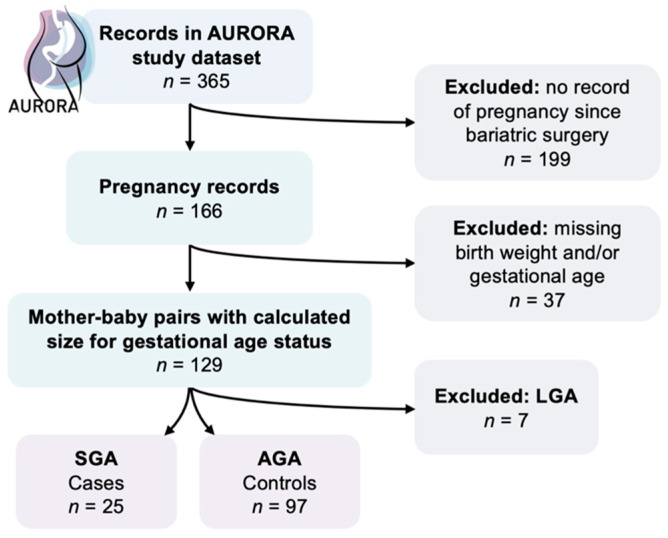
Inclusion of case and control babies in the AURORA dataset.

**Figure 2:**
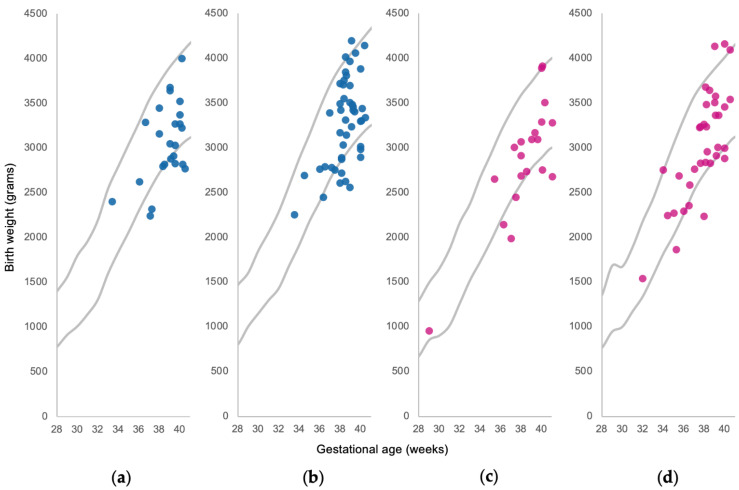
AURORA cohort babies plotted against 10th and 90th size for gestational age percentiles for birth weight, gestational age, infant sex, and parity in Flanders, Belgium using SPE data: (**a**) primiparous boys; (**b**) multiparous boys; (**c**) primiparous girls; (**d**) multiparous boys.

There were 129 singleton pregnancies in the AURORA dataset with complete data for infant sex, birth weight, and gestational age (Figure 1). These babies were plotted against size for gestational age percentiles for birth weight, gestational age, infant sex, and parity in Flanders, Belgium (Figure 2). Seven babies were above the 90th percentile and were excluded from the study. Twenty-five babies fell under the 10th percentile and became SGA cases. The remaining 97 babies between the 10th and 90th percentiles served as AGA controls. Many of the AGA babies fell closer to the 10th percentile than the 90th percentile, highlighting how there is a decrease in size for gestational age after bariatric surgery compared with the general birth population in Flanders.

Characteristics of the infants born from the AURORA cohort are detailed in Table 2. There were more boys than girls born in the cohort (56.6% vs. 43.4%, respectively). SGA babies had a lower mean birth weight than AGA babies (2594 g vs. 3185 g). There was no difference between gestational age of SGA and AGA babies (38.6 weeks vs. 38.3 weeks), and none of the babies were born post-term. A similar proportion of SGA and AGA babies were born pre-term (16% vs. 15.5%).

Maternal sociodemographic characteristics and clinical factors are shown in Table 3. Mothers of SGA and AGA babies were of a similar age (30 years) and height (164.1 cm vs. 165.8 cm). Less than a third of babies were their mothers’ first baby (31.2%) with the majority being born to parous mothers (63.1%), and the same proportions were seen for both SGA and AGA babies. There were no mothers of AGA babies with an underweight preconception BMI, and only two mothers of SGA babies (8%). There were fewer SGA mothers with obesity compared to AGA (28% vs. 33%). Out of the women with obesity, all but one in the SGA group had class I obesity, whereas in the AGA group, nearly half had class II obesity. Only one woman had class III obesity preconceptionally; in contrast, the majority of women had class III obesity prior to surgery (63.1%). There was little difference in pre-surgery BMI between mothers of SGA and AGA infants. All of the SGA mothers had a pre-surgery BMI in class II obesity or above, whereas 8% of the AGA mothers had an overweight or class I obesity BMI. A large proportion of ethnicity data was missing (41%); however, 88% of mothers with ethnicity data identified as White European, with similar proportions for both SGA and AGA mothers. Fewer mothers of SGA babies were married (36.1% vs. 12%), attended higher education (19.6% vs. 4%), or were employed (36.1% vs. 12%); however, 55% of mothers had missing data for these three variables. All of the SGA babies were conceived spontaneously whereas 14.4% of the AGA babies were conceived through fertility treatment, in contrast to what is expected, as fertility treatment is associated with low birth weight [22]. The majority of mothers in AURORA had RYGB surgery (73%), with a higher proportion in the SGA group (80% vs. 71.1%). LAGB was the second most common surgery type (15.6%) with slightly fewer carried out in SGA mothers (12% vs. 16.5%).

### 3.2. Modifiable Factors

**Figure 3:**
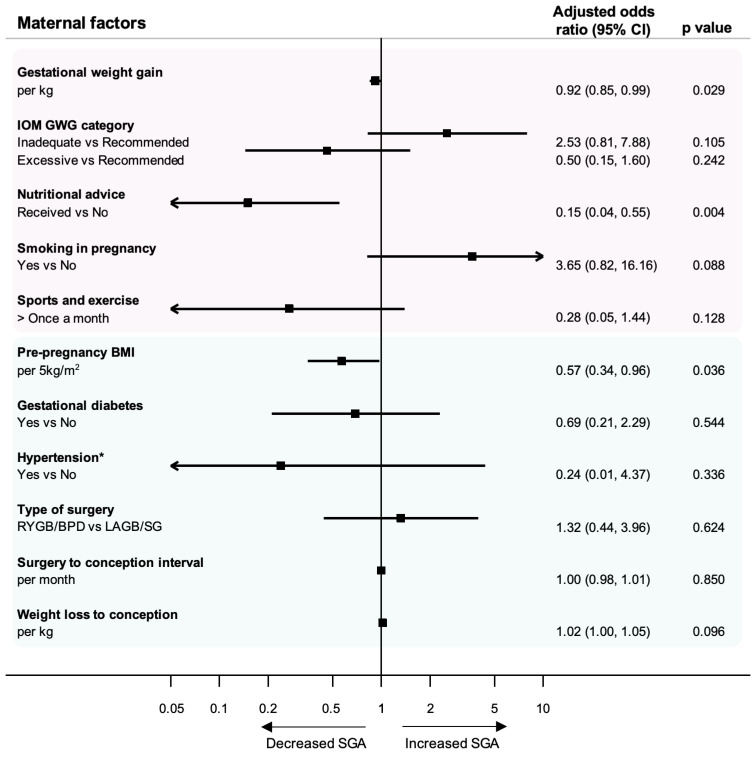
Age-adjusted analysis of maternal factors and their association with SGA. Modifiable factors are highlighted in pink, and clinical indicators are highlighted in blue. * The result for hypertension is a crude odds ratio (95% CI) due to zero cases in the SGA group.

The modifiable factors investigated in the AURORA mothers are detailed in Table 4, with the maternal age-adjusted analysis in Figure 3. Mothers of SGA babies had a significantly lower mean GWG at 9.8 ± 7.1 kg compared to AGA mothers at 13 ± 6.2 kg (*p* = 0.029) (Table 4). For every additional 1 kg gained during pregnancy, there was a significant decrease in odds of SGA (adjusted OR 0.92, 95% CI 0.85–0.99) (Figure 3). Only 27% of the mothers in AURORA had recommended GWG in pregnancy according to IOM recommendations, with 22.1% having inadequate GWG and 46.7% having excessive GWG. More than twice as many mothers of SGA babies had inadequate GWG compared with mothers of AGA babies (44% vs. 17.5%, respectively). In contrast, twice as many mothers of AGA babies had excessive GWG compared with mothers of SGA babies (28% vs. 50% respectively).

Less than half of women in AURORA reported receiving nutritional advice between bariatric surgery and pregnancy (47.5%). Women that reported receiving nutritional advice prior to pregnancy were significantly less likely to deliver an SGA baby compared with women who reported that they did not receive nutritional advice (adjusted OR 0.15, 95% CI 0.04–0.55, *p* = 0.004) (Figure 3). Over half of mothers of AGA babies reported receiving nutritional advice compared to under a third of SGA mothers (51.5% vs. 32%). Patterns were seen in the data for smoking and exercise in mothers of SGA and AGA babies, but the data did not find significant associations. More SGA mothers reported smoking during pregnancy compared to AGA mothers (16% vs. 6.2%). Fewer mothers of SGA babies took part in sports or exercise more than once a month in comparison to AGA mothers (8.0% vs. 22.7%).

The nutritional serum levels of mothers in AURORA are detailed in Table 5, alongside reference ranges specific for the second trimester of pregnancy [23]. Although women who received nutritional advice were significantly less likely to have an SGA baby, this study did not find evidence of any difference between serum levels of SGA and AGA mothers for the nutritional biomarkers investigated (Table 5). Several women in AURORA had lower than recommended levels of hemoglobin, iron, vitamin B12, and vitamin D. Mothers of SGA babies had slightly lower levels compared to AGA mothers of iron (81.1 vs. 90.1 μg/dL) and vitamin B12 (207.8 vs. 236.9 ng/L), but otherwise all mean values were similar between SGA and AGA mothers for the available data (missing data ranged from 10 to 72%).

### 3.3. Clinical Indicators

The clinical indicators investigated in the AURORA mothers are detailed in Table 6, with the maternal age-adjusted analysis presented in Figure 3. Mean preconception BMI of mothers of SGA and AGA babies in AURORA was 28.3 kg/m^2^ compared to the mean pre-surgery BMI of 43.2 kg/m^2^ (Table 6). There was a significant decrease in odds of SGA for every 5-unit increase in preconception BMI (adjusted OR 0.57, 95% CI 0.35–0.97, *p* = 0.036) (Figure 3). There were additional patterns observed in the data in the absence of statistical significance. There were fewer mothers of SGA babies that had gestational diabetes (16.0% vs. 24.7%) or pregnancy-induced hypertension (0% vs. 7.2%) compared to AGA mothers. More women of SGA babies had a malabsorptive type of surgery compared to mothers of AGA babies (80.0% vs. 74.2%). There was little difference between SGA and AGA mothers for time between surgery and conception, with a mean interval of over four years in both groups (48.9 vs. 50.0 months). Weight loss from surgery to conception was higher for mothers of SGA babies compared to AGA mothers (45.6 kg vs. 39.0 kg).

Sensitivity analyses showed that adjusting the analyses for maternal height did not change the effect size or direction for any factors (Table S2).

## 4. Discussion

The study aimed to identify potential risk factors of SGA to allow early identification of high-risk women or opportunities for intervention. Maternal bariatric surgery is a known risk factor for SGA, and this study demonstrated that 20% of the babies born in AURORA were SGA, which is double the rate of the general birth population. Women with lower GWG, lower preconception BMI, and those who reported not receiving nutritional advice prior to pregnancy were significantly more likely to have an SGA baby. Trends in the data show that fewer mothers of SGA babies took part in sports and exercise, and more SGA mothers smoked during pregnancy.

Lower GWG was significantly associated with an increase in odds of SGA, and more mothers of SGA babies had inadequate GWG compared to mothers of AGA babies. Half of the women in AURORA had excessive GWG yet still gave birth to a small or appropriate for gestational age baby. Excessive GWG has been shown to be associated with postpartum weight retention and weight regain following bariatric surgery [24]. This raises the question of what range of GWG is optimal for balancing health outcomes of both mother and baby and, until then, how women can be best supported to gain the recommended amount of weight.

There were a significant decrease in odds of SGA for women that reported receiving nutritional advice following bariatric surgery, highlighting the importance of health professionals in providing appropriate preconception and pregnancy-specific nutritional support [25]. Dietary advice provided should be appropriate for both post-bariatric surgery and pregnancy, such as an emphasis on a high lean protein intake as well as fruit and vegetables, and tailored on the basis of preconception BMI, GWG, and diagnosis of co-morbidities. For example, this includes limiting energy dense foods where excessive GWG is identified, and reducing rapidly absorbed carbohydrates for women with gestational diabetes [25].

This study could not find any evidence for a difference in the nutritional serum levels of mothers with SGA or AGA babies; however, the analyses were limited by a large amount of missing data for nutritional levels. The findings from this study highlight the importance of sufficient calorie intake after bariatric surgery, alongside adequate micronutrient monitoring. As the women in the AURORA cohort have regular nutrient monitoring, any deficiencies are picked up, and supplements are provided. A previous study in AURORA found that even though women were more likely to have a deficiency after bariatric surgery, supplementation during pregnancy allowed healthy levels to be restored, whereas a deficiency in the general obstetric population may not be picked up [26]. Future studies could explore a wider range of dietary biomarkers with a larger sample size.

Smoking was previously shown to be increased after bariatric surgery, and it was suggested that patients may use smoking or other forms of substance abuse to compensate for reduced food intake after surgery [27]. In our study, more mothers of SGA babies smoked during pregnancy compared to AGA mothers, and the adverse effects of smoking on fetal development and placental function are already well-established [28]. Although smoking during pregnancy is universally discouraged, this group of women may require targeted interventions to explore underlying reasons for smoking, especially if smoking commenced post-surgery. An important area which requires further study is placental function after bariatric surgery, as impaired nutrient transport across the placenta compromises fetal growth. This study showed that exercise during pregnancy may decrease the chances of having an SGA baby after bariatric surgery, which is in line with previous research demonstrating that increased physical activity improves placental vascularity and function, and subsequently nutrient and oxygen transfer, and additionally that mothers that exercise during pregnancy are more likely to have an AGA baby than either SGA or LGA babies [29,30,31].

Mothers of SGA babies in this study had a significantly lower preconception BMI, and fewer had obesity-related comorbidities such as hypertension and gestational diabetes compared to mothers of AGA babies. A maternal underweight status is associated with an increased risk of SGA in the general population; however, in our study, only two women had an underweight preconception BMI, and over two-thirds of the SGA mothers had a preconception BMI category of overweight or obesity [32]. This suggests that there may be underlying factors involved such as weight loss from bariatric surgery. In this study, SGA mothers lost more weight between bariatric surgery and conception compared to AGA mothers. Our study findings regarding gestational diabetes and hypertension were in contrast to the general obstetric population, where hypertensive disorders of pregnancy are associated with an increased risk of SGA [33]. As only a small percentage of women will experience gestational diabetes and/or hypertension during pregnancy, the sample size of this study was not large enough to be confident in the pattern observed. Further investigation is warranted to examine if factors that measure the success of bariatric surgery (e.g., excess weight loss and resolution of comorbidities) could be indicators of physiological changes that result in a high-risk subsequent pregnancy. This study found no difference in the surgery to conception interval; however, the intervals were long in this cohort with nearly 75% of women conceiving after 18 months. A previous retrospective study with a larger proportion of women (61%) becoming pregnant within 18 months of RYGB showed that a smaller interval makes inadequate GWG more likely, which suggests that there may be a higher likelihood of SGA babies with shorter surgery to conception intervals [34]. We could not address hypoglycemic events during pregnancy in this study; however, previous research indicates that hypoglycemia in pregnant women with a history of bariatric surgery occurring after the now-contraindicated OGTT for gestational diabetes may increase the risk of SGA [35].

The strength of this study is that it uses data from a unique, prospective cohort of women who are followed up for measurements before bariatric surgery, during preconception, during each trimester, and after delivery. The detail and number of variables recorded in AURORA are not routinely collected during antenatal care and therefore would not be possible to retrieve from a population database. The limitations include sample size and statistical power. Bariatric surgery patients make up a very small percentage of the obstetric population, and, subsequently, this study is limited in numbers, and many results have large confidence intervals. For some of the factors, there may have been a real and moderate effect, but insufficient results to allow for the drawing of reliable conclusions. For this reason, the results of this study focused on trends in the data and clinical significance in addition to statistical significance. However, this study does provide a valuable starting point for future research directions. As bariatric surgery increases, it is important that larger studies of subsequent pregnancies are conducted. Another limitation is that as the AURORA cohort methodology involved up to 11 follow ups, and part of the data was collected from self-administered questionnaires, this resulted in missing data for some variables due to non-attendance at follow up or item non-response in the questionnaires. Finally, many babies are born small due to hereditary factors such as having small parents, and this study was unable to differentiate between healthy small babies and babies that were born small due to a health complication such as malnutrition or growth restriction. However, the sensitivity analysis adjusting for maternal height showed no difference in effect size or direction for any factors’ association with SGA. Additionally, the average height of women in the AURORA study matched the average adult female height in Belgium of 165.5 cm [36].

## 5. Conclusions

As previously established, women with a history of bariatric surgery have an increased risk of delivering an SGA baby, especially for RYGB and BPD, which may in part be a result of inadequate nutrient absorption and caloric intake following these procedures [8]. This study found that higher GWG in pregnancy after bariatric surgery significantly reduces the odds of having an SGA baby. However, excessive GWG predisposes for weight regain postpartum [24]. Mothers that received nutritional advice post-surgery are also significantly less likely to have an SGA baby. As with all pregnancies, healthy behaviors such as regular exercise and avoiding smoking should be encouraged post-surgery. Women with a history of bariatric surgery should be provided with specialized support before and during pregnancy to ensure adequate nutritional intake and healthy fetal growth. GWG should be monitored throughout pregnancy, and growth scans are recommended every trimester at a minimum, or more frequently for women with additional risk factors to detect any potential restricted growth [25].

## Figures and Tables

**Table 1:** Institute of Medicine 2009 guidance for GWG in pregnancy [18].

Pre-Pregnancy BMI	Total Weight Gain Range (kg)
Underweight (<18.5 kg/m^2^)	12.5–18
Recommended (18.5–25 kg/m^2^)	11.5–16
Overweight (25–30 kg/m^2^)	7–11.5
Obesity (>30 kg/m^2^)	5–9

**Table 2:** Characteristics of babies born to mothers in AURORA.

Infant Characteristics	Total *n* = 122	SGA *n* = 25 (20.5%)	AGA *n* = 97 (79.5%)	*p* Value
**Sex**				
Boys	69 (56.6%)	15 (60.0%)	54 (55.7%)	0.697
Girls	53 (43.4%)	10 (40.0%)	43 (44.3%)	
**Birth weight**				
Mean ± SD	3064.1 ± 535.5	2594.4 ± 328.0	3185.2 ± 512.1	0.000
<2500 g	16 (13.0%)	9 (36.0%)	7 (7.2%)	0.002
2500–4000 g	103 (84.4%)	16 (64%)	87 (89.7%)	
≥4000 g	3 (2.5%)	0	3 (3.1%)	
**Gestational age**				
Mean ± SD	38.4 ± 1.9	38.6 ± 1.6	38.3 ± 2.0	0.696
<37 weeks	19 (15.6%)	4 (16.0%)	15 (15.5%)	1.000
37–42 weeks	103 (84.4%)	21 (84.0%)	82 (84.5%)	
≥42 weeks	0	0	0	

**Table 3:** Sociodemographic characteristics and clinical factors of mothers in AURORA.

Maternal Characteristics	Total *n* = 122	SGA *n* = 25	AGA *n* = 97	*p* Value
**Age at delivery, years** Mean ± SD	30.1 ± 4.6	30.4 ± 4.7	30 ± 4.5	0.727
**Height, cm** Mean ± SD	165.5 ± 6.9	164.1 ± 5.7	165.8 ± 7.2	0.267
**Parity**				
Nulliparous	38 (31.2%)	8 (32%)	30 (30.9%)	0.586
Multiparous	77 (63.1%)	13 (52%)	64 (66%)	
**Preconception BMI, kg/m^2^**				
Underweight <18.5	2 (1.6%)	2 (8%)	0	0.193
Recommended 18.5–24.9	34 (27.9%)	7 (28%)	27 (27.8%)	
Overweight 25.0–29.9	46 (37.7%)	9 (36%)	37 (38.1%)	
Obesity class I 30–34.9	24 (19.7%)	6 (24%)	18 (18.6%)	
Obesity class II 35–39.9	14 (11.5%)	1 (0.4%)	13 (13.4%)	
Obesity class III ≥ 40	1 (0.8%)	0	1 (1%)	
**Pre-surgery BMI, kg/m^2^**				
Overweight 25.0–29.9	1 (0.9%)	0	1 (1%)	0.477
Obesity class I 30–434.9	7 (5.7%)	0	7 (7.1%)	
Obesity class II 35–39.9	29 (23.8%)	8 (32%)	21 (21.6%)	
Obesity class III ≥ 40	77 (63.1%)	16 (64%)	61 (62.9%)	
**Ethnicity**				
White European	63 (51.6%)	13 (52%)	50 (51.5%)	1.000
Other	8 (6.6%)	1 (4%)	7 (7.2%)	
**Marital status**				
Married	38 (31.2%)	3 (12%)	35 (36.1%)	0.046
Single	29 (23.8%)	8 (32%)	21 (21.6%)	
**Education level**				
Higher education	20 (16.4%)	1 (4%)	19 (19.6%)	0.153
Secondary or below	47 (38.5%)	10 (40%)	37 (38.1%)	
**Employment status**				
Full or part-time work	38 (31.2%)	3 (12%)	35 (36.1%)	0.046
Unemployed	29 (23.8%)	8 (32%)	21 (21.6%)	
**Method of conception**				
Spontaneous	105 (86.1%)	25 (100%)	80 (82.5%)	0.039
Fertility treatment	14 (11.5%)	0	14 (14.4%)	
**Type of bariatric surgery**				
RYGB	89 (73.0%)	20 (80%)	69 (71.1%)	0.962
LAGB	19 (15.6%)	3 (12%)	16 (16.5%)	
SG	9 (7.4%)	2 (8%)	7 (7.2%)	
BPD	3 (2.5%)	0	3 (3.1%)	

Percentages do not add up to 100 in the case of missing data (Table S1).

**Table 4:** Analysis of maternal modifiable factors’ association with SGA.

Modifiable Factors	Total *n* = 122	SGA *n* = 25	AGA *n* = 97	Sig. Test *p* Value	OR (95% CI)	*p* Value
**GWG, kg** Mean ± SD	12.3 ± 6.5	9.8 ± 7.1	13.0 ± 6.2	0.023	0.92 (0.85–0.99)	0.029
**IOM GWG guidelines**						
Inadequate	27 (22.1%)	11 (44.0%)	16 (16.5%)	0.012	2.55 (0.82–7.93)	0.105
Recommended	33 (27.0%)	7 (28.0%)	26 (26.8%)		Reference group	-
Excessive	57 (46.7%)	7 (28.0%)	50 (51.5%)		0.52 (0.16–1.64)	0.265
**Nutritional advice**						
Yes	58 (47.5%)	8 (32.0%)	50 (51.5%)	0.004	0.14 (0.04–0.51)	0.003
No	13 (10.7%)	7 (28.0%)	6 (6.2%)		Reference group	-
**Smoking in pregnancy**						
Yes	10 (8.2%)	4 (16.0%)	6 (6.2%)	0.082	3.83 (0.88–16.69)	0.073
No	54 (44.3%)	8 (32.0%)	46 (47%)		Reference group	-
**Sports and exercise**						
More than once a month	24 (19.7%)	2 (8.0%)	22 (22.7%)	0.123	0.29 (0.06–1.49)	0.14
Once a month or less	38 (31.1%)	9 (26.0%)	29 (30.0%)		Reference group	-

Percentages do not add up to 100 in the case of missing data (Table S1). Significance test *p* values were calculated using *t* test, Mann-Whitney U test, Pearson’s χ2 test, or Fisher’s exact test. OR (95% CI) and subsequent *p* values are calculated from univariate logistic regression.

**Table 5:** Analysis of maternal nutritional biomarkers’ association with SGA.

Serum Levels	Reference Range *	AURORA Range (*N*)	SGA Mean ± SD	AGA Mean ± SD	*p* Value
**Haemoglobin** (g/dL)	9.7–14.7	8.7–13.9 (110)	11.3 ± 1.3	11.4 ± 1.1	0.851
**RBC count** (10^6^/μL)	2.81–4.49	3.12–4.94 (100)	3.87 ± 0.35	3.90 ± 0.34	0.777
**Iron** (μg/dL)	44–178	24–169 (49)	81.1 ± 41.0	90.1 ± 38.4	0.517
**Folate** (ng/mL)	0.8–24	4.4–19.7 (77)	15.9 ± 5.0	14.3 ± 5.1	0.245
**Vitamin B12** (ng/L)	130–656	77–760 (103)	207.8 ± 95.2	236.9 ± 110.6	0.232
**Calcium** (mmol/L)	2.05–2.25	2.08–2.39 (34)	2.22 ± 0.08	2.26 ± 0.08	0.276
**Vitamin D** **25-OH** (ng/mL)	10–22	4.5–68 (87)	33.2 ± 15.3	29.1 ± 13.3	0.373
**Albumin** (g/L)	25–45	29.8–41.4 (76)	36.8 ± 2.6	37.1 ± 2.3	0.951

* Reference ranges for serum levels specific to the second trimester of pregnancy [23]. RBC: red blood cell, 25-OH: 25-hydroxy. *p* values were calculated using t test or Mann-Whitney U test.

**Table 6:** Analysis of maternal clinical indicators’ association with SGA.

Clinical Indicators	Total *n* = 122	SGA *n* = 25	AGA *n* = 97	Sig. Test *p* Value	OR (95% CI)	*p* Value
**Preconception BMI, kg/m^2^**						
Mean ± SD	28.3 ± 5.1	26.4 ± 5.0	28.8 ± 5.1	0.066		
per 1 kg/m^2^ increase					0.90 (0.81–0.99)	0.038
per 5 kg/m^2^ increase					0.58 (0.35–0.97)	0.038
**Gestational diabetes**						
Yes	28 (23%)	4 (16%)	24 (24.7%)	0.541	0.69 (0.21–2.29)	0.543
No	77 (63.1%)	15 (60%)	62 (63.9%)		Reference group	-
**Hypertension**						
Yes	7 (5.7%)	0	7 (7.2%)	0.342	* 0.24 (0.01–4.37)	0.336
No	112 (91.8%)	24 (96%)	88 (90.7%)		Reference group	-
**Type of surgery**						
RYGB/BPD	92 (76.7%)	20 (80%)	72 (74.2%)	0.658	1.28 (0.43–3.79)	0.658
LAGB/SG	28 (23.3%)	5 (20%)	23 (23.7%)		Reference group	-
**Surgery to conception** **interval, months** Mean ± SD	49.8 ± 37.5	48.9 ± 34.0	50.0 ± 38.5	0.856	1.00 (0.99–1.01)	0.890
**Weight loss from surgery to conception, kg** Mean ± SD	40.4 ± 17.4	45.6 ± 14.4	39.0 ± 17.9	0.057	1.02 (1.00–1.05)	0.100

Percentages do not add up to 100 in the case of missing data (Table S1). Significance test *p* values were calculated using *t* test, Mann-Whitney U test, Pearson’s χ2 test, or Fisher’s exact test. OR (95% CI) and subsequent *p* values are calculated from univariate logistic regression. * Hypertension OR (95% CI) was calculated by substituting zero cases to 0.5.

## Data Availability

All data analyzed in this study are provided in the aggregated form in the relevant tables throughout the article. The raw AURORA study data are not publicly available due to ethics and privacy.

## Supplementary Materials

The following are available online at https://www.mdpi.com/article/10.3390/nu13051699/s1, Table S1: Missing data in the analysis dataset, Table S2: Sensitivity analyses examining the effect of maternal height on the association between maternal factors and SGA.
