# Supplementary material for: Pregnancy after Bariatric Surgery: A Nested Case-Control Study of Risk Factors for Small for Gestational Age Babies in AURORA
Source: Nutrients. 2021 May 17;13(5):1699. doi: 10.3390/nu13051699 (PMC8156275; doi:10.3390/nu13051699)
Supplement: Supplementary file 1 [file Table S1. docx.pdf › Table S1. docx.pdf]

Supplementary Material: Pregnancy after bariatric surgery: a nested case-control study of risk factors for small for gestational age babies in *AURORA*

Table S1. Missing data in the analysis dataset

	**Total**<br>n = 122	**SGA**<br>n = 25	**AGA**<br>n = 97
**Infant sex**	0	0	0
**Birth weight**	0	0	0
**Gestational age**	0	0	0
**Age at delivery**	0	0	0
**Height**	1 (0.8%)	0	1 (0.8%)
**Parity**	7 (5.7%)	4 (16.0%)	3 (3.1%)
**Preconception BMI**	1 (0.8%)	0	1 (0.8%)
**Pre-surgery BMI**	8 (6.6%)	1 (4.0%)	7 (7.2%)
**Ethnicity**	51 (41.8%)	11 (44.0%)	40 (41.2%)
**Marital status**	55 (45.1%)	14 (56.0%)	41 (42.3%)
**Education level**	55 (45.1%)	14 (56.0%)	41 (42.3%)
**Employment status**	55 (45.1%)	14 (56.0%)	41 (42.3%)
**Method of conception**	3 (2.6%)	0	3 (3.1%)
**Type of bariatric surgery**	2 (1.6%)	0	2 (2.1%)
**GWG**	5 (4.1%)	0	5 (5.1%)
**Nutritional advice**	51 (41.8%)	10 (40.0%)	41 (42.3%)
**Smoking**	58 (47.5%)	13 (52.0%)	45 (46.4%)
**Sports and exercise**	60 (49.1%)	14 (56.0%)	46 (47.4%)
**Haemoglobin**	12 (9.8%)	3 (12.0%)	9 (9.3%)
**RBC count**	22 (18.0%)	7 (28.0%)	15 (15.5%)
**Iron**	73 (59.8%)	15 (60.0%)	58 (59.8%)
**Folate**	45 (36.9%)	12 (48.0%)	33 (34.0%)
**Vitamin B12**	19 (15.6%)	6 (24.0%)	13 (13.4%)
**Calcium**	91 (74.6%)	19 (76.0%)	72 (74.2%)
**Vitamin D 25-OH**	35 (28.7%)	8 (32.0%)	27 (27.8%)
**Albumin**	46 (37.7%)	12 (48.0%)	34 (35.1%)
**Gestational diabetes**	17 (13.9%)	6 (24.0%)	12 (12.4%)
**Hypertension**	3 (2.5%)	1 (4.0%)	2 (2.1%)
**Surgery to conception interval**	4 (3.3%)	0	4 (4.1%)
**Weight loss to conception**	8 (6.6%)	1 (4.0%)	7 (7.2%)
