# Supplementary material for: Pregnancy after Bariatric Surgery: A Nested Case-Control Study of Risk Factors for Small for Gestational Age Babies in AURORA
Source: Nutrients. 2021 May 17;13(5):1699. doi: 10.3390/nu13051699 (PMC8156275; doi:10.3390/nu13051699)
Supplement: Supplementary file 1 [file Table S2. docx.pdf › Table S2. docx.pdf]

Supplementary Material: Pregnancy after bariatric surgery: a nested case-control study of risk factors for small for gestational age babies in *AURORA*

Table S2. Sensitivity analyses examining the effect of maternal height on the association between maternal factors and SGA

Maternal factors	Crude result OR (95% CI)	p value	Maternal age adjusted OR (95% CI)	p value	*Sensitivity analyses* Maternal age and height adjusted OR (95% CI)	p value
**Gestational weight gain, per kg**	0.92 (0.85-0.99)	0.029	0.92 (0.85-0.99)	0.029	0.91 (0.85-0.98)	0.028
**IOM GWG guidelines**						
Inadequate	2.55 (0.82-7.94)	0.105	2.53 (0.81-7.88)	0.109	2.65 (0.84-8.37)	0.096
Recommended	Reference group	-	Reference group	-	-	-
Excessive	0.52 (0.16-1.64)	0.265	0.50 (0.15-1.60)	0.242	0.52 (0.16-1.69)	0.277
**Nutritional advice**						
Yes	0.14 (0.04-0.51)	0.003	0.15 (0.04-0.56)	0.005	0.11 (0.03-0.49)	0.004
No	Reference group		Reference group		Reference group	
**Smoking in pregnancy**						
Yes	3.83 (0.88-16.69)	0.073	3.69 (0.83-16.39)	0.086	3.84 (0.86-17.23)	0.079
No	Reference group	-	Reference group	-	Reference group	-
**Sports and exercise**						
More than once a month	0.29 (0.06-1.49)	0.140	0.28 (0.05-1.44)	0.128	0.21 (0.04-1.20)	0.080
Once a month or less	Reference group	-	Reference group	-	Reference group	-
**Pre-pregnancy BMI**						
per 1kg/m^2 change	0.90 (0.81-0.99)	0.038	0.89 (0.81-0.99)	0.036	0.90 (0.81-1.00)	0.043
per 5kg/m^2 change	0.58 (0.35-0.97)	0.038	0.57 (0.34-0.96)	0.036	0.59 (0.35-0.98)	0.043
**Gestational diabetes**						
Yes	0.69 (0.21-2.29)	0.543	0.69 (0.21-2.30)	0.549	0.66 (0.20-2.20)	0.498
No	Reference group	-	Reference group	-	Reference group	-
**Hypertension**						
Yes	0.24 (0.01-4.37)*	0.336	-	-	-	-
No	Reference group	-	-	-	-	-
**Surgery to conception interval, per month**	1.00 (0.99-1.01)	0.890	1.00 (0.99-1.01)	0.844	1.00 (0.99-1.01)	0.940
**Type of surgery**						
RYGB/BPD	1.28 (0.43-3.79)	0.658	1.31 (0.44-3.94)	0.627	1.39 (0.46-4.21)	0.564
LAGB/SG	Reference group	-	Reference group	-	Reference group	-
**Weight loss to conception, per kg**	1.02 (1.00-1.05)	0.100	1.02 (1.00-1.05)	0.096	1.02 (1.00-1.05)	0.100

*Hypertension OR was calculated using 0.5 substitution due to zero count in case group therefore no adjustments were made
